# Unicorns transforming the practice of urology: value creation and allocation in the digital age

**DOI:** 10.1007/s00345-025-05828-6

**Published:** 2025-07-16

**Authors:** Philipp Erben, Severin Rodler, Amin T. Turki, Ulrich Witzsch, Christian Thielscher

**Affiliations:** 1 Department of Urology and Urosurgery, Medical Faculty Mannheim, Heidelberg University, Theodor-Kutzer Ufer 1-3, 68167 Mannheim, Germany; 2 Department of Urology, University Hospital Schleswig- Holstein (UKSH), Campus Kiel, Kiel, Germany; 3 Department of Hematology and Oncology, Marienhospital University Hospital, Ruhr-University Bochum, Bochum, Germany; 4 Institute for AI in Medicine, Universitätsklinikum Essen, Essen, Germany; 5 Chair of Workgroup on IT and Documentation in Urology, German Society for Urology, Dusseldorf, Germany; 6 Urological Clinic, Sulzbach Ts, Germany; 7 Competence Center for Medical Economics, FOM University, Chair, Essen, Germany; 8 DGfMS (Deutsche Gesellschaft für Medizinsysteme - German Society for Medical Systems), Solingen, Germany

**Keywords:** Urology, Unicorns, Value, Allocation, Artificial intelligence, Molecular testing

## Abstract

**Purpose:**

To analyze what kind of products and services unicorns, which create a substantial financial value, provide in urology and what this means for the future of urological practice. So far, the added value of unicorns has hardly been utilised for medical research, although a fraction of it would be enough to multiply the funds for urological research.

**Methods:**

All medical unicorns (as by end of 2024) were studied, and urological unicorns identified. Publicly available data on valuation, investors, business operations and geographic distribution were analyzed to determine entrepreneurial purpose and value creation in urology.

**Results:**

Among the 21 unicorns in urology, the majority operate in drug research, including artificial intelligence guided development; this is in line with the digital transformation of medicine. Yet, business models are diversified. The valuations range from $ bn 1.0 to 7.83, summing to a total value of $ bn 56.2. The overwhelming majority (90%) is based in the US, one in China and one in UK.

**Conclusion:**

Unicorns in urology have accumulated substantial financial impact, which may accelerate medical progress but also creates risks (such as creating inequity). These developments will likely impact the practice of urology and deserve more scientific research to inform medical societies and policy decision making.

## Introduction

Unicorns summarize a group of young companies (mainly startups) that are currently not listed on the stock exchange but are already worth more than one billion US dollars. They create substantial financial value in a short period of time. At the start of the year 2025, the domains of healthcare and life sciences include over 100 unicorns with a total value of almost 270 billion US dollars [1, 2]. By way of comparison, this figure corresponds almost exactly to the expenditure of the entire statutory health insurance system in Germany in 2022, covering 70 million lives, which amounted to 266 billion euros [3].

Unicorn companies are typically only 5–15 years old, however, there are even “faster” unicorns: a few companies achieved unicorn status in less than one year [4]. Around 1% of all seed-financed start-ups (i.e. start-ups financed by investors in the early stages) make it to unicorn status [5].

The sheer amount of value generated by unicorns could have a massive impact on medicine. Firstly, it may redirect financial flows, which can lead to the strengthening of some participants, but also to the weakening of others. The innovations that emerge in new companies could change treatment patterns. Finally, the money that is currently absorbed by unicorns will have to be paid back at some point and may extract money from (some) health care providers.

Health care as a whole is a complex system, even more so when taking economic aspects into account; focusing on one specialty often enables more meaningful analysis [6]. Therefore, this article focuses on unicorns in urology—which is also the specialty most readers of this journal are most interested in. In addition, many diseases in this field cause massive burden of disease [7]. Conditions such as benign prostatic hyperplasia (BPH), urinary tract infections, incontinence, urinary stones, prostate and bladder cancer are of rising prevalence with growing challenge for the health care systems, both medically and financially [8–10]. These diseases also drive costs by increasing demand for services in the elderly, creating further challenges to public health policies and healthcare systems worldwide, and a potential shortage of urologists.

Finally, urology develops rapidly, especially in new diagnostic procedures, treatments, information technology, and digitalization [7, 11]—in alle these fields, health unicorns prevail.

The main aim of our investigation is to achieve a better understanding what kind of products and services urological unicorns provide, how much value they create, and what this means for the future of urology.

## Materials and methods

All 118 unicorns in the field of healthcare and life science in the list of CB Insights (2024) were analyzed to determine the extent to which they are urologically significant [12].

We included all companies which are either.

- Dedicated to urological diseases (e.g., if a company is focused on treatment of lower urinary tract symptoms), and / or.
- Provide new diagnostic or therapeutic methods or improve research in urology (including urologic oncology).

In this latter case, we included only companies that investigate diagnostics or drugs that will cure urological diseases; that is, we excluded companies active only in non-urological areas. If in doubt (e.g., if a company researches drugs of which it is unclear yet whether they can be used for urological diseases), we included the company in our analysis. The same holds true for companies that facilitate patient recruiting for (urological) studies.

We excluded companies that are only marginally involved in urological diagnosis, treatment or research; for example, insurance companies (even if they cover—amongst others—costs of urological diseases). We also excluded companies focused on diabetes treatment (although, obviously, renal failure is a typical second disease).

For the selected companies, i.e. those active in the field of urology, we analyzed in more detail which products and/or services they offer to patients, doctors and others and categorized this as their “business model”. The analysis was based on publications by the companies themselves, particularly on the internet, and publications by third parties. The respective websites were searched, with a focus on the business purpose and business figures, and the company name was searched for in MEDLINE, Google Scholar, and standard search engines.

The categorization of a company’s main business model was based on several, iterative rounds of assigning main activities to the companies so that the categories became complete and disjoint (i.e., mutually exclusive and collectively exhaustive).

## Results

We identified 21 unicorns involved in urology (a detailed list, including a description of the respective business areas, is provided in Table 1). Their valuations range from $ bn 1.0 to 7.83; their total value sums up to $ bn 56.2. The companies’ geographic distribution is almost exclusively concentrated in the US (90.5%, *n* = 19), one company is registered in China, and one in UK.

**Table 1:** List of urological unicorns. (source: CB insights 2024 [12], own research)

Company name	Country	Value (USD bn)	Description	Business model
*Caris*	United States	7,83	Caris focuses on molecular science, specifically in the domain of cancer care. The company offers services such as molecular profiling, blood profiling, and tissue profiling, which provide comprehensive molecular information to help oncologists create personalized treatment plans for cancer patients. It primarily sells to the healthcare industry, particularly oncology. It was founded in 1996 and is based in Irving, Texas.	Molecular science services
*Benchling*	United States	6,1	Benchling is a cloud-based platform focused on biotechnology research and development within the biotech sector. The company offers a suite of software tools designed to enhance scientific data management, collaboration, and insights for R&D processes. It was founded in 2012 and is based in San Francisco, California.	Platform services for R&D
*Color*	United States	4,6	Color specializes in genetic testing and health services, focusing on early detection and management of cancer and hereditary diseases. The company offers genetic screenings to assess the risk for hereditary cancer and heart conditions, as well as services to understand medication responses, aiming to facilitate preventative health measures and personalized care plans. Color primarily serves employers, health plans, unions, and clinicians, providing them with tools to manage the health of their populations. Color was formerly known as Color Global. It was founded in 2014 and is based in Burlingame, California.	Genetic testing
*Abogen*	China	3,7	Abogen operates as a plant-based pharmaceutical research company. It focuses on the research and development of messenger ribonucleic acid (mRNA) drugs to create antidotes against human disease. The company was founded in 2019 and is based in Suzhou, China.	Pharmaceutical research
*Komodo Health*	United States	3,3	Komodo Health specializes in healthcare analytics and operates within the healthcare technology sector. The company offers a platform that provides insights by analyzing a range of healthcare data, aiming to improve patient care and reduce disease burden. Komodo Health primarily serves life sciences companies, healthcare practitioners, payers, and patient advocacy groups with its suite of software applications designed to deliver value in healthcare through data-driven insights. It was founded in 2014 and is based in San Francisco, California.	Health data analytics
*Eikon Therapeutics*	United States	3,2	Eikon Therapeutics is a biopharmaceutical company focused on the discovery and development of novel treatments for life-threatening diseases. The company offers a platform that integrates engineering, science, and advanced microscopy to visualize and measure protein movement in living cells, aiding in the identification of new drug targets and the development of therapeutics. Eikon’s pipeline includes programs in oncology, immunology, and neuroscience. It was founded in 2019 and is based in Hayward, California.	Pharmaceutical research
*CMR Surgical*	United Kingdom	3	CMR Surgical specializes in the development of surgical robotics, focusing on the medical devices industry. Its flagship product, Versius, is a modular and portable surgical robot designed to facilitate minimal access surgery, seamlessly integrating into existing hospital workflows without the need for infrastructure changes. CMR Surgical primarily serves the healthcare sector, with a particular emphasis on enhancing the capabilities of surgical teams and improving patient outcomes through advanced robotics and data-driven insights. CMR Surgical was formerly known as Cambridge Medical Robotics. It was founded in 2014 and is based in Cambridge, United Kingdom.	Robotics
*Somatus*	United States	2,5	Somatus focuses on value-based kidney care. The company offers integrated care services for patients with or at risk of developing kidney disease, utilizing technology and clinical services to delay or prevent disease progression, decrease hospital utilization, improve quality and care coordination, and increase the use of home dialysis modalities and kidney transplantation rates. Somatus primarily serves the healthcare sector, partnering with health plans, health systems, nephrology, and primary care groups. It was founded in 2016 and is based in McLean, Virginia.	Integrated kidney care
*insitro*	United States	2,44	Insitro focuses on drug discovery and development. The company’s main services involve the use of machine learning and high-throughput biology to predict successful paths for medicine creation. It aims to avoid costly failures in pharmaceutical research and development (R&D). Insitro primarily sells to the healthcare industry. It was founded in 2018 and is based in South San Francisco, California.	Pharmaceutical research
*Freenome*	United States	2,62	Freenome serves as a biotechnology company that focuses on early cancer detection through advanced diagnostic tools. The company develops blood tests that identify early signs of cancer by analyzing biomarkers from tumor and non-tumor sources using a multiomics platform. Its tests are designed to be non-invasive and accessible, aiming to detect various types of cancer at its most treatable stages. It was founded in 2014 and is based in South San Francisco, California.	Cancer blood tests
*Xaira Therapeutics*	United States	2,15	Xaira Therapeutics focuses on revolutionizing drug research and development within the biopharmaceutical industry through the use of artificial intelligence. The company offers a platform designed to enhance the drug discovery process, aiming to streamline and improve the efficiency of developing new therapeutics. Xaira Therapeutics primarily serves the biopharmaceutical sector with innovative solutions to accelerate drug R&D. It was founded in 2023 and is based in San Francisco, California.	AI support for pharmaceutical research
*Medable*	United States	2,1	Medable specializes in providing digital clinical trial software solutions within the healthcare and pharmaceutical sectors. The company offers a comprehensive platform that facilitates the management of clinical trials, including tools for remote data collection, electronic consent (eConsent), patient-reported outcomes (ePRO), and clinical outcome assessments (eCOA), all designed to streamline the trial process and enhance data quality. Medable was formerly known as Dermatrap. It was founded in 2012 and is based in Palo Alto, California.	AI support for clinical studies
*ConcertAI*	United States	1,9	ConcertAI is a leader in clinical artificial intelligence and real-world data products within the healthcare sector. The company offers a suite of AI-driven solutions and services designed to optimize clinical trials, provide predictive patient insights, and support commercial healthcare solutions. ConcertAI primarily serves the oncology research and clinical development sectors. It was founded in 2017 and is based in Cambridge, Massachusetts.	AI support for clinical studies and health data analytics
*Cambrian BioPharma*	United States	1,79	Cambrian BioPharma operates as a clinical-stage drug development company focused on the biomedicine sector. The company’s main offerings include the development of therapeutics designed to prevent diseases and lengthen healthspan. Cambrian BioPharma primarily serves the healthcare industry. It was founded in 2019 and is based in New York, New York.	Pharmaceutical research
*Tessera Therapeutics*	United States	1,69	Tessera Therapeutics is a life sciences company with a focus on genetic medicine and biotechnology. The company’s main service is Gene Writing, a new genome engineering technology that writes therapeutic messages into the genome to treat diseases at their source. Tessera Therapeutics primarily serves the healthcare and medical research sectors. It was founded in 2018 and is based in Cambridge, Massachusetts.	Pharmaceutical research
*Generate Biomedicines*	United States	1,36	Generate Biomedicines is a therapeutics company that operates at the intersection of machine learning, biological engineering, and medicine. The company’s main offerings include the development of novel medicines with specific therapeutic functions using a platform that applies learned rules from the study of proteins. Generate Biomedicines primarily serves the biopharmaceutical industry with its drug discovery and development processes. It was founded in 2018 and is based in Somerville, Massachusetts.	Pharmaceutical research
*Orna Therapeutics*	United States	1,5	Orna Therapeutics is a biotechnology company that focuses on the development of fully engineered circular RNA (oRNA) therapeutics, a new class of RNA medicines. The company’s main offerings include the creation of oRNAs that can realize the full potential of RNA and change the way diseases are treated. These oRNAs have applications across multiple disease areas including cancer, regenerative medicine, protein replacement, infectious diseases, and autoimmunity. It was founded in 2019 and is based in Cambridge, Massachusetts.	Pharmaceutical research
*Everly Health*	United States	1,3	Everly Health is a digital health platform specializing in diagnostics-driven care and the healthcare sector. The company offers at-home testing kits and digital tools for diagnosing and managing recurring health conditions, providing results online. Everly Health primarily serves the healthcare ecosystem, including individual consumers and enterprise clients. Everly Health was formerly known as Everly Well. It was founded in 2015 and is based in Austin, Texas.	Home test kits and AI-driven diagnostics
*Visby Medical*	United States	1,12	Visby Medical develops polymerase chain reaction (PCR) based diagnostic tests for the detection of infectious diseases. The company offers medical solutions for sexual health tests, respiratory health tests, and other infectious diseases. It was formerly known as Click Diagnostics. The company was founded in 2012 and is based in San Jose, California.	PCR-based diagnostics
*Orca Bio*	United States	1	Orca Bio is a clinical-stage biotechnology company focused on developing high precision allogeneic cell therapy products for the healthcare sector. The company’s main offerings include proprietary therapeutic mixtures of immune and stem cells intended to replace diseased blood and immune systems with healthy ones, aiming to transform allogeneic cell therapy with better outcomes and fewer risks. It was founded in 2016 and is based in Menlo Park, California.	Pharmaceutical research
*Owkin*	United States	1	Owkin operates as an artificial intelligence (AI)-based biotechnology company. It utilizes AI to enhance the process of finding suitable treatments for patients. The company’s main services include the use of AI to identify new treatments, accelerate clinical trials, and build diagnostic tools. It enables researchers in hospitals, universities, and the biopharmaceutical industry to understand how drug efficacy varies for the improvement of drug development. Its services primarily cater to the biopharmaceutical and academic research sectors. It was founded in 2016 and is based in New York, New York.	AI support for pharmaceutical research

In contrast to the geographical distribution, the business models are more diverse, with 12 main business models these unicorns are engaged in. The most common business model of these unicorns is pharmaceutical research, underlying the relevance of innovative drug design to the advance of medical practice and the substantial improvements in urology and uro-oncology (Table 2).

**Table 2:** Main company business activities of unicorns in urology

Business model	Number of unicorns
Pharmaceutical research	8
AI support for pharmaceutical research	2
AI support for clinical studies and / or health data analytics	2
Cancer blood tests	1
Genetic testing	1
Health data analytics	1
Home test kits and AI-driven diagnostics	1
Integrated kidney care	1
Molecular science services	1
PCR-based diagnostics	1
Platform services for R&D	1
Robotics	1

The investment background is diverse. CB Insights (2024) lists three main investors per unicorn [12]. Despite the concentration in value within individual companies, no business conglomerates have been observed among unicorns. Only six of the main investors are represented at more than one company (five investors are involved in two companies, and one investor in three companies). Most investors only appear once on the list.

During the research, it became clear as an incidental finding that urological unicorns also.

- Grow very quickly by “burning” substantial investments, for example by using considerable financial resources for product development and/or marketing, which do not come from their own sales but are contributed by investors, and.
- They diversify their business activities as quickly as possible, i.e. they quickly add other products in addition to those with which they started (e.g. a company starts with AI/gene-based molecule development and then buys gene sequencing laboratories).

## Discussion

Across several areas of businesses, unicorns create enormous value; accordingly, research in this field has dramatically increased in the last year [13]. Whilst unicorns in urology are valued at approx. $ 60 bn, research activities to better understand their relevance and impact have just started (to our knowledge, this is the first publication on urological unicorns).

Given the relevance of financial funds flowing into medical research, unicorns potentially accelerate medical progress [14]. Our analysis shows that by far the prevailing business model of urological unicorns is pharmaceutical research, which is in line with the overwhelming activities of established pharmaceutical companies to medical value creation worldwide. However, it is striking that the urologic unicorns have strongly involved Artificial Intelligence (AI) in the process of drug design, which is one relevant element of the digital transformation of medicine [15]. However, as for other medical disciplines [16] urological oncology is still at the beginning of transformative processes to involve AI in its practices, whereas radiology is leading the development of AI enabled devices in clinical trials [17]. Hence, its impact of unicorns on urological practice and care is more difficult to estimate nowadays. Due to its innovative character requiring drug development and clinical testing, most of their research projects are still in the “pipeline”—not yet in clinical practice. In the course of the next few years, it will become possible to calculate and quantify the significance of unicorn investments in terms of medical progress, and their efficiency (how good are unicorns in turning research money into tangible medical results? ).

Whilst providing chances through value creation and research, unicorns may also produce some risks; for example, they may change the organization of medicine—its “management”—and this does not automatically lead to an improvement in the lives of everyone involved (for example, if roles and / or distribution of power change) [18]. Some side-effects aren’t easy to predict: the introduction of new telemedicine services, e.g., can lead to traditional doctors treating more difficult cases with reduced budgets; [19] the privatization of research associated with unicorns can lead to a decline in publicly available research results [20].

Because most of the unicorns in urology are involved in pharmaceutical research with a focus on artificial intelligence (AI), some of the potential risks are related to AI and big data itself. These include the generation and spread of disinformation, the growth of health inequity, cybersecurity issues, and patient safety risks [21]. Especially in the health system, (dis-)information, equity, and safety are of specific relevance. Furthermore, risks can depend on complex interactions of technologies with the social, political and economic context in which they are deployed. These risks have rarely been studied yet but need to be specifically analyzed [22].

The overwhelming majority of the unicorns studied is based in the US, although some authors found profitability to be higher in Europe [23]. However, this finding is not limited to urology but general to investment in medicine. There are a few European companies with unicorn status in Europe, e.g. Doctolib from France, an appointment scheduling and telemedicine platform, currently worth USD 6.4 billion. It seems that European companies struggle with reaching unicorn status in urology. There may be a variety of reasons for this finding, some which can be only speculated about.

First, the availability of financial funds seems to be much better in the US as compared to the EU. For unicorns in general, the European commission found a more developed venture capital ecosystem in the US: [24] about 50% of investors in European unicorns are of non-European origin, with 77% based in the US. US investors may also be more eager to invest heavy sums whereas EU investors prefer slow, “organic” growth. Cash flow of a typical unicorn is still negative after 5 years (see, e.g., Yahoo finance 2024) [25].

As a side note, with regards to urological unicorns, we found that investors are “spread” across unicorns, with few being invested in more than one of them. There are several health-related investors (e.g., Orbis, Andreesen Horowitz) and some big pharmaceutical companies such as Astellas and Roche. We simply don’t know yet why this is the case and why lead investors change from company to company.

A further reason for the lack of European unicorns might be the fragmentation of the European market. In fact, there is still one market per country for marketing purposes, at least from the standpoint of language, reimbursement, and regulation. For example, the European Union has 24 official languages [26].

Urological and uro-oncological research may be more intensive in the US; and the US market for urological products may be bigger and / or more profitable and faster growing. Future research will find better insights into these issues.

It is unclear whether the current valuations of urological unicorns transfer into stable, positive cash-flows in the future. This can be analyzed only in a few years by following up on the development of unicorns. In earlier research [27] it was found that about 60% of value increase goes to investors, and only 1% to clinicians. Whether this holds true for urological unicorns is currently unknown.

There are more questions that deserve further investigation, e.g.: how is the relationship between investors and urologists? Where are urological unicorns created (e.g., as spin-off from universities? ). Which unicorns grow into long-term improvements in medical care, and which ones don’t—and why?

Health policymakers should continue to monitor what happens to the enormous value generated by unicorns: does the value created ultimately support medical care, or does it flow away, e.g., by withdrawing money from health care in order to pay off investors? Awareness of the public is appropriate, and regulatory measures may be required.

## Data Availability

No datasets were generated or analysed during the current study.
